# Myoelectric Pattern Recognition Outperforms Direct Control for Transhumeral Amputees with Targeted Muscle Reinnervation: A Randomized Clinical Trial

**DOI:** 10.1038/s41598-017-14386-w

**Published:** 2017-10-23

**Authors:** Levi J. Hargrove, Laura A. Miller, Kristi Turner, Todd A. Kuiken

**Affiliations:** 1 Center for Bionic Medicine, Shirley Ryan AbilityLab, Chicago, IL 60610 USA; 2 Department of Physical Medicine and Rehabilitation, Northwestern University, Chicago, IL 60610 USA; 3 Department of Biomedical Engineering, Northwestern University, Evanston, IL 60208 USA

## Abstract

Recently commercialized powered prosthetic arm systems hold great potential in restoring function for people with upper-limb loss. However, effective use of such devices remains limited by conventional (direct) control methods, which rely on electromyographic signals produced from a limited set of muscles. Targeted Muscle Reinnervation (TMR) is a nerve transfer procedure that creates additional recording sites for myoelectric prosthesis control. The effects of TMR may be enhanced when paired with pattern recognition technology. We sought to compare pattern recognition and direct control in eight transhumeral amputees who had TMR in a balanced randomized cross-over study. Subjects performed a 6–8 week home trial using direct and pattern recognition control with a custom prostheses made from commercially available parts. Subjects showed statistically better performance in the Southampton Hand Assessment Procedure (p = 0.04) and the Clothespin relocation task (p = 0.02). Notably, these tests required movements along 3 degrees of freedom. Seven of 8 subjects preferred pattern recognition control over direct control. This study was the first home trial large enough to establish clinical and statistical significance in comparing pattern recognition with direct control. Results demonstrate that pattern recognition is a viable option and has functional advantages over direct control.

## Introduction

Amputation of the arm at or above the elbow constitutes a significant disability that can adversely affect life quality^1^. People use their arms and hands for a diverse range of activities, from touching and manipulating objects in their environment to interacting with each other through gestures and non-verbal communication. Current prosthetic limbs, whether they are body-powered or myoelectric, remain poor substitutes for a natural arm. Body-powered devices require harnesses to capture mechanical movements from the users and can only restore limited joint movements or degrees-of-freedom (DOFs). Myoelectric control systems allow the user to operate a prosthetic limb by measuring and decoding electromyographic (EMG) signals, which are generated by residual limb muscle contractions. Historically, upper limb amputees use the difference in the EMG signals from a single pair of antagonistic muscles to control a single DOF at a time; this is often referred to as direct control. For transhumeral amputees, electrodes are placed over the biceps and triceps to control a prosthetic elbow. Controlling a hand requires the amputee to use a slow and inconvenient switching mechanism. Controlling a third DOF, such as a wrist, requires yet another switch and becomes quite cumbersome. Recently commercialized powered devices offer great potential in further restoring lost function^2^, but remain sorely limited by these conventional recording methods.

We developed a novel surgical procedure called targeted muscle reinnervation (TMR) to provide improved myoelectric control^3–5^. In this surgical procedure, severed nerves from an amputated limb are transferred onto residual limb target muscles that no longer have biomechanical function because of the amputation. Target muscles are first denervated so that they can be reinnervated by transferred residual arm motor nerves that previously controlled arm and hand function. Reinnervated target muscles serve as biological amplifiers of the motor nerve commands intended for the missing arm and thus provide physiologically appropriate EMG control signals related to arm/hand function, making prosthesis control more intuitive. For example, after reinnervation of a segment of biceps muscle by the transferred median nerve, contraction of that muscle—as the user attempts to close his or her missing hand—generates EMG signals that provide control input to the prosthesis to close the motorized hand; and conversely, reinnervation of a segment of triceps by the transferred distal radial nerve generates EMG signals that control opening.

TMR has been a very successful procedure with 23 out of 26 of the first TMR recipients of surgeries performed at Northwestern University and San Antonio Military Medical successfully operating a TMR-controlled prosthesis^6^. The procedure is growing in popularity and is performed clinically to provide improved control of prosthetic limbs and to prevent neuroma pain^7^. The reinnervated muscles contain rich neural information that corresponds to the amputated limb function, including information for the intrinsic muscles of the hand. Conventional amplitude decoders are unable to extract this information. EMG pattern recognition systems have shown great promise, but only in offline studies and limited laboratory demonstrations thus far^4,8,9^. Pattern recognition measures EMG signals from a set of residual limb muscles and uses machine learning algorithms to learn patterns from physiologically appropriate muscle contractions. Consequently, there is no need for a switching mechanism, resulting in seamless sequential control of multiple degrees-of-freedom. Pattern recognition myoelectric control systems have now been successfully commercialized by Coapt, LLC (Drs. Hargrove and Kuiken have a financial interest in Coapt, LLC, a company that sells pattern recognition myoelectric control systems. No Coapt products or materials were used in this study). Here, our objective was to present a comprehensive laboratory and home trial to evaluate functional improvements provided by TMR and pattern recognition for individuals with transhumeral amputation. To the best of our knowledge, this is the first study to compare the effects of direct and pattern recognition control in subjects who received the TMR procedure.

## Results

### Subject Recruitment

**Figure 1:**
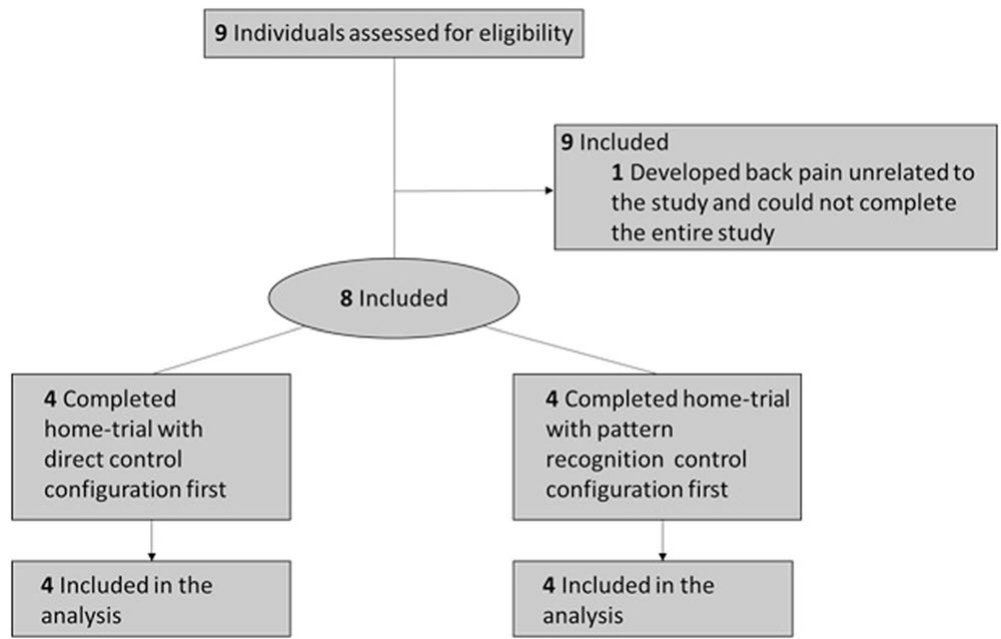
Schematic of randomized block design.

**Figure 2:**
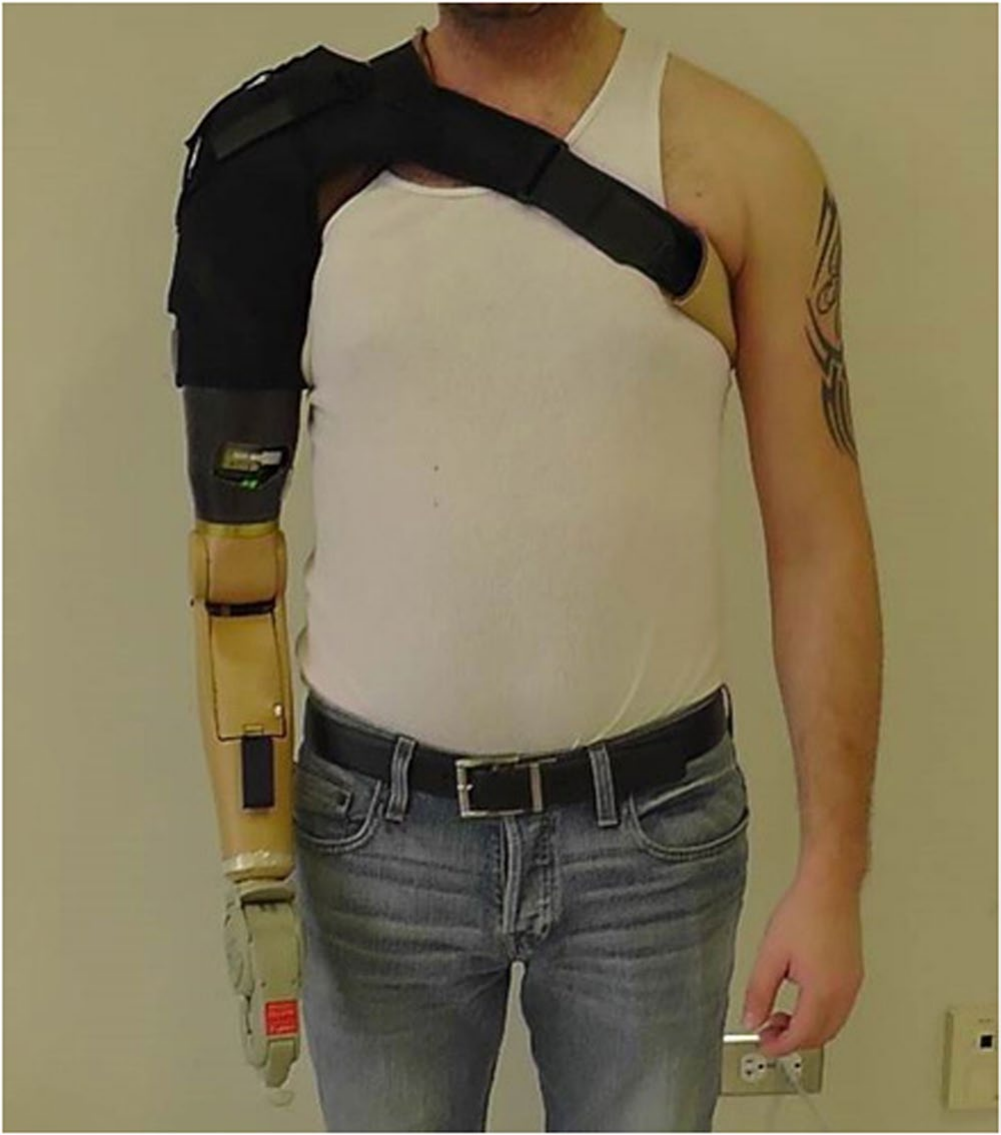
Representative subject wearing the physical prosthesis with a Greifer terminal device (Ottobock, Inc.).

Nine males with transhumeral amputations who had undergone the TMR procedure provided informed consent and were recruited for the study; however one subject withdrew due to back pain unrelated to the study methods (Fig. 1, Table 1). All participants were competent myoelectric prosthesis users prior to enrolling in the study. Each participant was issued a prosthesis that was custom fabricated from commercially available parts (see Methods). Participants could choose between using either a powered split-hook (electric terminal device or electric Greifer terminal device) or a single degree-of-freedom hand, depending on their preference and experience. All prostheses had a motorized elbow, wrist, and terminal device (Fig. 2). Seven subjects chose to use a powered hook terminal device and one participant chose to use a myoelectric hand for his terminal device. Participants agreed to wear their prostheses using both direct control and pattern recognition configurations for a minimum six-week home trial. Using a balanced randomized block design^10^, four participants were selected to use the conventional direct control configuration first, and four were selected to use the pattern recognition control system first (see Methods).

**Table 1:** Subjects enrolled in study.

Subject	Age (years)	Time since amputation (years)	Time since TMR (years)	Side	Gender	Etiology	Terminal Device used	Control test order
S1	35	4	3	R	M	Trauma (military)	Hook-ETD	PR-DC
S2	45	2	1	R	M	Trauma (train)	Hand	DC-PR
S3	54	6	<1	L	M	Trauma (military)	Hook-ETD	DC-PR
S4	58	5	1	L	M	Sarcoma	Hook-ETD	PR-DC
S5	25	6	6	L	M	Trauma	Hook-ETD	DC-PR
S6	31	8	7	L	M	Trauma (military)	Hook-Greifer	PR-DC
S7	27	2	1	R	M	Trauma (crushing)	Hook-Greifer	DC-PR
S8	31	1	1	R	M	Trauma (MVA)	Hook-ETD	PR-DC

*ETD with passive wrist unit.

### In Lab Testing with Physical Prosthesis

**Figure 3:**
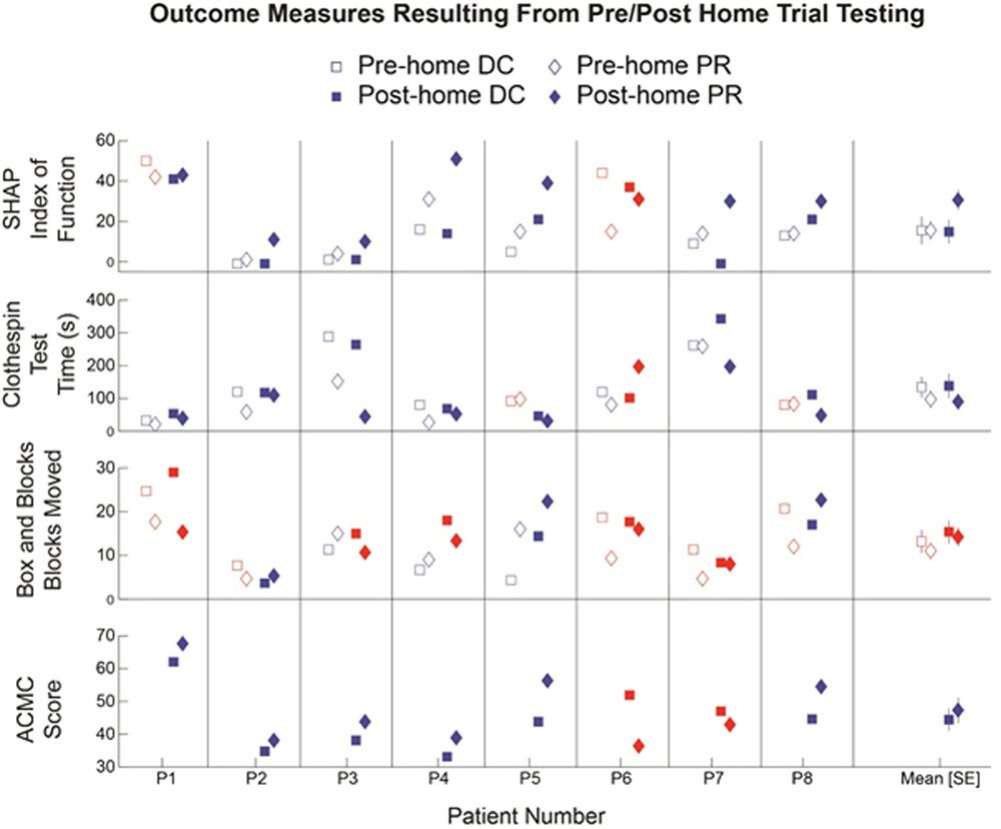
Summary of outcomes testing using the physical prosthesis. Hollow markers denote pre-home trial testing and solid markers denote post-home testing. Blue markers show data points indicating more favorable performance with pattern recognition, and red markers show data points indicating more favorable control with direct control.

Before and after each home trial the following tests were performed: the Southampton Hand Assessment Procedure (SHAP) in which the subject must grasp a variety of objects^11^; the Clothespin Relocation Task where subjects must move three clothespins from a horizontal to a vertical pole^12^; the Box and Blocks Test where subjects must move one block from a box over a 4-inch wall to another box^12^; and (post-trial only) the Assessment for Capacity for Myoelectric Control (ACMC)^13^, a validated observational test of the subject performing functional tasks. Scores for each test are summarized for each subject in Fig. 3.

#### SHAP

**Figure 4:**
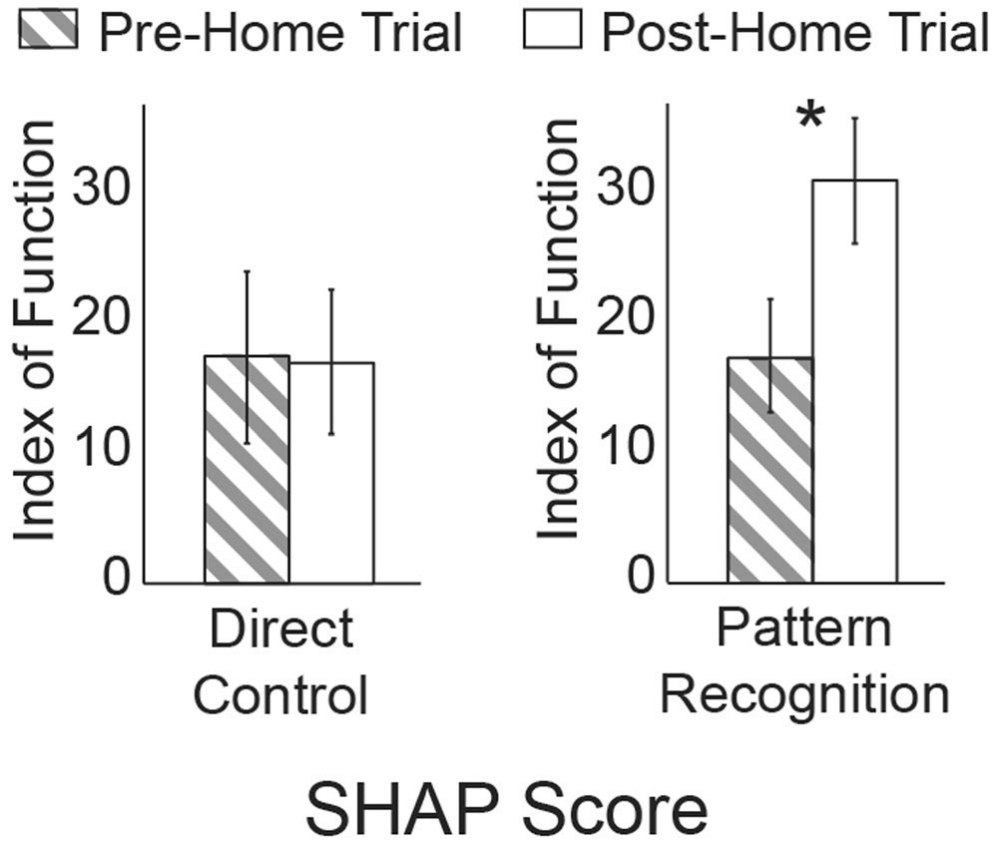
Examination of the SHAP index of function between pre and post home trial testing to help interpret the statistically significant interaction term.

Subjects performed significantly better (higher scores) when performing the SHAP using pattern recognition compared to using direct control (p = 0.041). There was also a significant interaction between pre- and post-home trial test results and the control system used to complete the test (p = 0.038). Upon further investigation of the data, we noticed no change in index of function (IOF) the SHAP between pre and post testing with direct control by a large increase in the IOF between pre and post testing when using pattern recognition control (Fig. 4). Our interpretation of these results is that the subject learned to use the pattern recognition control system over the course of the home-trial to better complete the activities associated with the SHAP.

#### Clothespin Relocation Task

Subjects performed significantly better (lower scores) when performing the Clothespin Relocation Task using pattern recognition control compared to using direct control (p = 0.024). At the end of the trial, subjects moved three clothespins from a horizontal bar to a vertical bar in 90.2 ± 39.6 seconds using pattern recognition compared to 137 ± 60.2 seconds using direct control. There were no statistically significant changes between pre- and post-home trial testing, nor were there any significant interaction terms between pre/post home trials testing and the control strategy used.

#### Box and Blocks Test

There were no significant differences for the Box and Blocks Test, nor were there any changes between pre- and post-home trial testing. At the end of the home trial, subjects moved 13.4 ± 2.6 blocks using pattern recognition control and 15.6 ± 2.7 blocks using direct control.

#### ACMC Testing

There were no significant differences between ACMC test scores between pattern recognition and direct control. At the end of the home trial, subjects scored 47.3 ± 3.9 using pattern recognition and 44.4 ± 3.4 using direct control. The ACMC is one of a few validated outcome measures for use with upper-limb prosthetics with published minimally detectible change (MDC) of 2.5 units when one rater is used for the ACMC 3.0^14^. It is noteworthy that all subjects demonstrated a change in performance greater than this MDC, with six subjects showing more favorable performance (higher score) with pattern recognition and two subjects showing more favorable performance using direct control.

### Quantitative Home Usage Statistics Measured by the Control System

During the home trials, we monitored home usage over the duration of the trials Table 2. We measured the date and time that the prosthesis was powered on, and the commands that were sent by the control system to the prosthesis. On average, users cumulatively wore the prosthesis 138.7 ± 34.6 hours during the direct control portion of the home trial and 147.7 ± 45.3 hours during the pattern recognition phase of the home trial, although these wear times were not significantly different. Subjects choose to recalibrate their control on 32.6 ± 8.2 occasions over the duration of the home trial and the recalibration behavior was highly subject dependent.

**Table 2:** Wear time, recalibration and control preference.

Subject	Direct Control Wear Time (hrs)	Pattern Recognition Wear time (hrs)	Number of Recalibrations	Preference of Control
S1	41	15	7	PR
S2	280.1	301.6	39	PR
S3	196.8	183.6	73	PR
S4	254.6	366.9	56	PR
S5	91.4	85.1	10	PR
S6	54.9	27.9	20	DC
S7	157.7	128.5	18	PR
S8	33.2	73.0	38	PR

### Qualitative Home Usage Logs Maintained by the Subjects

Subjects were able to use both types of control to complete a variety of activities such as preparing food, eating, dressing, shopping, and performing household chores. The frequency with which they reported in their journal that they wore the prosthesis qualitatively correlated with the wear time recorded by the embedded control system. Regardless of the type of control system being used, there were a variety of factors which prevented them from using the prosthesis more. For example some individuals found the prosthesis to be heavy or too warm to wear on hot days.

### Qualitative Rating of Control

By the end of the trial, seven of the eight subjects reported that they preferred using pattern recognition control over direct control. Most subjects found performing direct control mode-switching to be inconvenient. Subjects also noted that sometimes the prosthesis moved unintentionally using pattern recognition control. However, subjects also noticed that sometimes it was hard to control only one-degree of freedom when desired using direct control, as simultaneous control of two-DOFs was always available. These unintentional movements, regardless of whether it occurred using pattern recognition or direct control, made the control less reliable for the subject.

## Discussion

Providing robust and reliable control systems for upper-limb amputees is an important patient and clinician identified need to improve user satisfaction and reduce abandonment of upper-limb myoelectric devices^15^. This is especially important for transhumeral amputees who require functional restoration of the elbow, wrist and hand degrees freedom. Over the past several years, we have developed two important improvements to address control limitations in this population: 1) TMR, and 2) pattern recognition control. TMR has received wide-spread clinical adoption due to the low risks of the procedure and clear functional advantages using only simple additional control strategies^12,16^. In limited controlled laboratory testing, pattern recognition has shown great promise to provide even further functional improvements^4,9^. The goal of this work was to complete a home trial with comprehensive outcomes testing to determine the effectiveness of TMR in combination with pattern recognition when used with commercially available physical prostheses. To the best of our knowledge, this is the first home trial to compare pattern recognition and direct control methods in subjects who have had TMR, and to demonstrate statistical significance. The subjects had TMR in hospitals across the country, and we specifically chose to evaluate both control methods using prostheses that were fabricated from commercial parts, furthering the viability of this research to be implemented with many current prosthetic hand and arm systems.

Subjects showed statistically significant improvements using pattern recognition for the SHAP and Clothespin Relocation scores, particularly after the completion of the home trial. This suggests that subjects learned to use the pattern recognition prosthesis over the duration of the home trial. Completing the activities of daily living associated with the SHAP requires movement of the elbow, wrist and the terminal device^17^ and competing the Clothespin Relocation Test also requires repeated movements of all 3-DOFs. It is likely that users performed better on these timed tests using pattern recognition because it allows for seamless sequential control without mode switching. Another contributing factor could have been the nature of the proportional control available to the user. Pattern recognition controls the velocity of movement by considering EMG signals from all of the electrodes rather than just from a single antagonistic muscle pair. As a result, more information is available to control slow and precise movements. There were no significant differences between the control strategies when considering the Box and Blocks Test. Successfully moving blocks only requires flexion and extension of the elbow and opening and closing of the terminal device. Consequently no mode switching was necessary. We believe that this is the primary reason that there were no significant differences between control types for this outcomes measure. In fact, we would not have been surprised if direct control would have performed better, because users had the opportunity to simultaneously operate the elbow and terminal device, which could have resulted in faster movements. We found no significant differences between control types when evaluating the ACMC scores, though six of the eight subjects did perform better using pattern recognition. The ACMC is not a timed test and thus it is possible that this could have had an impact across control comparisons. In hindsight, we would have completed the ACMC prior to the home trial to improve the power of our statistical test; however time constraints prevented this from being feasible.

Limited prior testing data is available from transhumeral TMR amputees. In a previous case series, three transhumeral TMR subjects completed the Box and Blocks Test and Clothespin Relocation Task using direct control^12^. Their performances on both of these outcome measures fell within the range of values reported in this study. We have also previously tested four transhumeral TMR amputees using a prosthetic limb that was tethered to a desktop computer and capable of performing either pattern recognition or direct control within a lab environment^18^. In this prior work, we found significant improvements in both the Clothespin Relocation Task and the Box and Blocks Test using pattern recognition. Further investigation found near identical performance on the Box and Blocks Test when using pattern recognition control. However, the prior study found that fewer blocks could be moved using a direct control system. The previously reported study required users to wear a hand rather than a terminal device of their choice, which in the present study was an Electric Terminal Device (ETD). This may have contributed to the discrepancy.

Pattern recognition has been proposed as an alternative to direct control for decades^19^, with most studies characterizing performance in terms of offline control metrics such classification error-rates. Numerous barriers to clinical deployment of pattern recognition systems have been cited including performance deterioration caused by changes in electrode positioning^20^, changes in residual limb positioning^21^, and changes in force variation. However, it is not clear if these offline performance metrics are sufficient to characterize online control performance where the user may incorporate real-time feedback to change their generated patterns^22^. The performance of pattern recognition has also been questioned because it does not naturally allow for simultaneous and independent control estimates. Our current study is important because in spite of all of these perceived limitations, pattern recognition control still have functional advantages over direct control, and was a viable control alternative in a home-environment. Other control methods, such as using continuous-based regression may provide additional benefits such as allowing simultaneous proportional control^23^ or promote more advantageous co-adaption between user and control system^24^, but these remain to be tested in a home environment.

Seven of the eight subjects indicated that they preferred pattern recognition control over direct control, with the exception of one. This subject performed poorer on all outcome measures using pattern recognition control, so it is unsurprising that he preferred direct control. The consistent reason that subjects provided for preferring pattern recognition was that mode switching was not required, and that they did not have to concentrate on making isolated contractions with their agonist/antagonist muscle pairs. Rather, they only had to make the physiologically appropriate contraction that was used during the training of the pattern recognition system.

All subjects wore their prosthesis at home to perform a variety of tasks. Common examples included completing household chores, cooking, shopping, and gardening. Nearly as insightful were the reasons that subjects chose not to wear the prosthesis. These reasons did not explicitly include poor control of the device, but rather other factors such as the weight of the device, the battery-life of the device, the temperature, having shoulder pain, performing water activities or being sun-burned.

## Methods

The experimental methods were approved by the Northwestern University Institutional Review Board (IRB). All methods were performed in accordance with the relevant guidelines and regulations stipulated in the IRB approval letter and no changes to important changes to study methods were made following approval. The informed consent document contained a detailed description of all study methods, including a provision for publication of identifying images in an online open-access scientific journal. The study design was a randomized crossover-study and was conducted between February 2013 to February 2016 at the Shirley Ryan AbilityLab (formerly the Rehabilitation Institute of Chicago). Participants were assigned to begin with direct control or pattern recognition control through a balanced and non-blinded block randomization administered by the prosthetist by drawing numbers from a concealed container. To be eligible for the study, subjects were required to be experienced myoelectric prosthesis users prior to enrolling, but were not required to be actively using their prostheses on a routine basis.

The surgical method has previously been described in detail^3,5^. Six different surgeons performed the TMR surgeries at hospitals across the United States. Briefly, the subjects received general anesthesia with no paralytic agents so that nerves could be identified easily with stimulation. An incision was made between the two heads of the biceps. The subcutaneous fat was dissected from distal to proximal and saved as a fat flap. The plane between the long and short head of the biceps was identified, widened, and explored to find the musculocutaneous and median nerves. The musculocutaneous nerve to the short head of the biceps was cut as it entered the muscle and the distal segment was buried in the long head so that it did not reinnervate the long head. Next the median nerve was identified and freed distally. It was then cut so that the proximal segment could be transferred to the short head motor point and the median nerve was simply sewn over the small motor point on to the muscle. The fat flap was then laid between the short and long heads of the biceps as a physical spacer that helped to separate the EMG signals once the recovery was complete and the subject was refit with a prosthesis using TMR. Essentially this same procedure was next done to the triceps so that the distal radial nerve innervating extensor muscles below the elbow was transferred to the lateral triceps and a fat flap separated the lateral triceps from the long head and medial heads of the triceps. After surgery it took about 12 weeks to see early reinnervation. Fitting of the TMR prosthesis was delayed until after six months so that the TMR muscles were well re-innervated and stable.

A custom fabricated prosthesis was created for each subject using commercially available parts: a Boston Digital Elbow (Liberating Technologies Inc.), a Motion Control Wrist Rotator (Motion Control Inc.), and a single degree-of-freedom terminal device of their choice (Fig. 1, and Table 1). Consequently the prosthesis was capable of performing the following powered movements: elbow flexion (EF), elbow extension (EE), wrist pronation (WP), wrist supination (WS), terminal device open (TDO), terminal device close (TDC), and no movement (NM). Many of the terminal devices also incorporated passive wrist flexion and extension. Each subject was fit with two custom fabricated silicone gel liners (Alps Inc.). Stainless steel electrodes were embedded into the wall of the liner and stretchable conductive fabric transmitted the EMG signals to the distal end of the liner. For the pattern recognition control liner, electrode locations were not targeted over specific muscles, rather a grid of electrodes was used as described in previous work^25^. For the direct control liner, four electrode locations were manually identified using a combination of surgical notes when available, palpation, and myoelectric signal testing^7^. At the distal end of the liners, the signals were amplified and digitized using a Texas Instruments ADS1299 chip sampled at 1000 Hz and transmitted to an embedded controller. The embedded controller could be reconfigured using software to allow for direct or pattern recognition control. Therefore, during the two phases of the study, the device, socket, and subject interface was unchanged, with the exception of the liner where the electrodes locations were moved. All other changes were done electronically. The decoded commands were then sent to the prosthesis and were logged in memory so that home-usage statistics could be determined at the end of the home trial. The amplifier gains were set on a subject specific basis with a typical value of 2000, and data were digitally filtered between 70–450 Hz. A recalibration switch was laminated into the outer wall of each socket so that the users could initiate a pattern recognition calibration routine whenever they desired. After being fit with the prosthesis, subjects received intensive occupational therapy and functional use training^26^. These sessions were spread out over three or four consecutive days that lasted approximately eight hours per day.

During the direct control phase of the study, subjects were fit with their prosthesis and adjustment to the control was made as necessary. The clinical procedure to configure a myoelectric control system for TMR recipients was used for each subject. The TMR surgery results in four spatially separated independent myoelectric control sites that may be used for control; the natively innervated biceps muscle, the natively innervated triceps muscles and biceps muscle reinnervated by the median nerve, and the triceps muscle reinnervated by the distal radial nerve. Electrodes were placed over these myoelectric control sites, as it would be done in a clinical environment. A dual-site differential direct control system was manually configured using each antagonistic muscle pair^7^. Typically these four control sites would be used to control elbow flexion and extension and terminal device opening and closing. To allow for the wrist rotation degree of freedom to be controlled, mode switches were configured for each subject according to their preference and previous device use. Subjects were allow to request recalibration of their direct control settings before completing the set of pre and post outcome measures, but could not change their direct control settings while at home during the trial.

Use of pattern recognition algorithms has been suggested as an alternative to direct control for decades^19^. Generally when using pattern recognition, EMG signals are divided into windows, represented by suitable features, and then classified using a mathematical model. Numerous studies have investigated each of these parameters and how they interact, and a review has been provided by Scheme and Englehart^21^. After the movement has been selected using pattern recognition, the speed of actuation is estimated using a non-linear combination of the EMG signal amplitudes^27^ and smoothed with a filter^28^. The embedded pattern recognition system used in this study has been described in detail in previous work which was tested with transradial amputees in a case series^29^.

Individuals took the device home for a minimum of 42 days (6 weeks) in each configuration. Users were instructed to use the prosthesis to complete activities of daily living and to keep a journal detailing how often they used the prosthesis and for what activities it was being used. If the prosthesis needed to be returned for repair or if the user had a valid and documented reason for not wearing a myoelectric prosthesis, then additional time was added to the home-trial to ensure that they had six weeks of usage. Examples of valid reasons to not wear the prosthesis included taking a beach vacation, being sunburned, etc.

Outcomes were measured prior to and after each home trial. The outcome measures included: the Southampton Hand Assessment Protocol (SHAP), the Jebsen-Taylor Test of Hand Function, the Box and Blocks Test, and the Clothespin Relocation Task. These measures were selected, in part, based on the recommendations of the American Academy of Orthotists and Prosthetists State of the Science meeting on Upper Limb Prosthetic Outcome Measures^30^. These measures were also chosen to evaluate hand, wrist and elbow function and were activities that could be reasonably completed with a physical prosthesis. Finally, at the end of the study, subjects were asked which type of control they would choose to use as part of a definitive prosthesis.

The main outcome measure in this study was a comparison in performance of the SHAP, Clothespin Relocation Test, Box and Blocks Test, and Jebson-Taylor Test of Hand Function. The secondary outcome measures was a change in performance between pre and post home trial scores. For all outcome measures except the ACMC, a two-way repeated measures analysis of variance (ANOVA) was completed on each outcome metric where subject was a random factor; time of testing condition (pre/post home trail) was a fixed factor and control condition (pattern recognition/direct control) were fixed factors. Non-normal data were transformed using a box-cox transformation was used prior to statistical testing. The interaction between time of testing and type of control used was also included in the model. Significance was tested at the α = 0.05 level. For the ACMC a paired t-test was used. An exploratory analysis was performed to analyze how long the prosthesis was used over the duration of the home-trial, which degrees of freedom were being controlled and at which speed.

### Data Availability

The datasets generated during and/or analyzed during the current study are available from the corresponding author on reasonable request.

### Clinical Trail Information

This is a registered clinical trial published on March 30, 2017: NCT03097978 https://clinicaltrials.gov/ct2/show/NCT03097978?term=NCT03097978&rank=1.
